# Existing and Potential Therapies for Post-Traumatic Stress Disorder and Persistent Post-Concussion Symptoms in Intimate Partner Violence: A Narrative Review

**DOI:** 10.3390/brainsci16040398

**Published:** 2026-04-08

**Authors:** Charlotte Copas, Abigail D. Astridge, Jennifer Makovec Knight, Stuart J. McDonald, Sandy R. Shultz, Georgia F. Symons

**Affiliations:** 1 Department of Neuroscience, School of Translational Medicine, Monash University, 99 Commercial Road, Melbourne, VIC 3004, Australia; charlotte.copas@monash.edu (C.C.); abigail.astridgeclarke@monash.edu (A.D.A.); jen.makovecknight@monash.edu (J.M.K.); stuart.mcdonald@monash.edu (S.J.M.); 2 Centre for Trauma and Mental Health Research, Health Sciences and Human Services, Vancouver Island University, Building 491, 900 Fifth Street, Nanaimo, BC V9R 5S5, Canada; 3 Institute of Aging and Lifelong Health, Faculty of Health, University of Victoria, Victoria, BC V8N 5M8, Canada

**Keywords:** intimate partner violence, psychotherapy, exercise, post-traumatic stress disorder, mild traumatic brain injury, persistent post-concussion symptoms

## Abstract

**Highlights:**

**What are the main findings?**

- Trauma-focused psychotherapies show promise in alleviating post-traumatic stress disorder (PTSD) symptomology in victim-survivors of intimate partner violence (IPV), while emerging exercise, mindfulness, and pharmacotherapy interventions require further validation.
- Despite the prevalence of IPV-related brain injuries, there is a lack of therapeutic studies specific to persistent post-concussion symptoms (PPCS). The translation of evidence from other populations with high rates of brain injury may help to inform new treatments for this cohort.

**What are the implications of the main findings?**

- Future interventions should move beyond a “one-size fits all” approach and integrate trauma-informed, financially accessible, and tailorable techniques to overcome systemic barriers that frequently prevent survivors from accessing traditional care.
- Developing integrated protocols that address the unique intersection of PTSD and PPCS may be essential for remediating chronic symptoms.

**Abstract:**

**Background:** Intimate partner violence (IPV) is a pervasive medical concern affecting millions of people worldwide, with the majority being women. IPV is linked to a number of long-term physical and mental health consequences, including brain injuries and associated persistent post-concussion symptoms (PPCS) and post-traumatic stress disorder (PTSD). Despite the high prevalence of these conditions, there is sparse literature assessing accessible and effective therapeutic avenues specific to IPV victim-survivors. **Methods:** This narrative review had two aims: to identify therapeutic studies addressing PTSD and PPCS in women IPV survivors, and to provide a narrative overview of potential therapeutic categories, including psychotherapy, mindfulness and meditation, exercise, and pharmacotherapy. A comprehensive literature search was conducted using PubMed and Google Scholar. Inclusion criteria required full-text, peer-reviewed articles published in English, conducted in women with a history of IPV, reporting treatment outcomes related to PTSD or PPCS. Where no IPV-specific evidence was identified, findings from closely related populations including military veterans, athletes, and general TBI samples were narratively reviewed to inform potential therapeutic implications. **Results:** Nineteen studies addressing PTSD in women IPV survivors were identified, predominantly utilizing psychotherapeutic or mindfulness and meditation-based interventions. No intervention studies targeting PPCS specifically in IPV survivors were identified. Consequently, results for PPCS are largely extrapolated from adjacent populations. Although potential therapeutic avenues were narratively identified across psychotherapy, mindfulness and meditation, exercise, and pharmacotherapy, IPV-specific evidence remains limited, and validation for PTSD and PPCS in this population is needed before clinical recommendations can be made. **Conclusions:** While 19 studies identified promising therapeutic options for IPV-related PTSD, no IPV-specific PPCS interventions were identified, and implications for PPCS management remain largely inferential. Validation and integrated trauma-informed approaches addressing the intersection of PTSD and PPCS are needed for this understudied population.

## 1. Introduction

Intimate partner violence (IPV) is a serious and understudied medical concern, defined as acts of physical and sexual violence (e.g., slapping, beating, forced sexual intercourse), emotional and psychological abuse (e.g., name-calling, death threats), and controlling behaviours (e.g., financial control) from current or former intimate partners [1]. IPV is experienced across genders but is most common in women, affecting 1 in 3 globally [2]. However, the true prevalence of IPV is likely underestimated, as stigma and lack of social, financial, and legal support often lead to underreporting [3]. IPV is linked to a number of long-term consequences of both physical and mental health.

Injuries to the head, face, and neck as a consequence of physical attacks have been overlooked despite prevalences ranging between 35% to 100% [4]. Consequent IPV brain injuries (IPV-BI), resulting from mild traumatic brain injuries (mTBI; concussion), and non-fatal strangulation (NFS) events are common among this population and are often repetitive and comorbid [5], leading to unique long-term health consequences [6,7,8]. Outside the context of IPV, 15% to 30% of individuals who experience mTBI will endure (e.g., >4 weeks) a constellation of symptoms that include somatic, mood, and cognitive impairments after the insult, commonly termed persistent post-concussion symptoms (PPCS) [9,10,11,12,13]. IPV-BI has been associated with greater concussion-like symptom burden [14,15,16,17,18]. While the exact prevalence of PPCS within this population is unknown, victim-survivors are at a high risk due to comorbid factors commonly associated with PPCS (i.e., female sex, chronic stress, prior head injuries and multiple traumas, psychiatric disturbances and alcohol abuse) [19].

Psychological violence often occurs in tandem with physical violence. As such, victim-survivors are also at high risk for developing a variety of psychopathologies [20] such as post-traumatic stress disorder (PTSD) which affects up to 64% of victim-survivors [21,22]. Compared with women without IPV exposure, survivors are 2.3 times more likely to develop PTSD and remain at substantially higher risk for PTSD, depression, and chronic pain years after abuse has ended [23,24]. In addition, IPV survivors are more likely to adopt risky coping behaviours such as smoking, excessive drinking, and isolation from their community [25]. This population also faces an increased risk for heightened feelings of depression, anxiety, borderline personality disorder, antisocial personality disorder, substance use disorders, and suicide ideation [4,26,27], with many occurring comorbidly [28].

The complex relationship between PTSD and TBI has been investigated in military contexts, where brain injury and traumatic experiences often occur together and therefore may translate to the setting of IPV [29,30]. Among veterans, combat-related mTBI is associated with an approximately two-fold increase in PTSD risk, and PTSD is the strongest predictor of persistent post-concussion symptoms even after controlling for symptom overlap [31]. Similar patterns are reported in IPV victim-survivors with TBI, who show greater PTSD severity and higher diagnostic rates than those without brain injury [32]. PPCS and PTSD may therefore be synergistically linked within an IPV setting where repeated trauma and chronic stress are present.

The mechanisms driving the bidirectional relationship between PTSD and TBI are yet to be fully elucidated. Early evidence suggests that white matter damage, due to brain injury, could disrupt limbic, neuroendocrine, and autonomic nervous system regulation. As such, an individual’s vulnerability to PTSD symptoms such as hypervigilance and exaggerated fear and defensive behaviours may be increased [33,34]. A few studies have identified white matter [35] and connectivity [36] changes in IPV survivors with a history of brain injury. While these studies controlled for PTSD symptoms, the regions where changes were identified overlap with those seen in PTSD studies [37,38].

Conversely, PTSD may also have implications on TBI outcomes. Persistent stress can contribute to neuroinflammatory process (i.e., overactivation of microglial expression, oxidative stress, and impaired glutamate neurotransmission), which deteriorate synaptic integrity, especially in the amygdala, pre-frontal cortex, and hippocampus [39]. Indeed, imaging studies have shown that veterans with PTSD have reduced cortical thickness [40] and hippocampal anatomical and functional abnormalities [41], even when controlling for TBI status [42]. As such, dysregulation of neuroinflammatory mechanisms and physical alterations in specific brain regions are pathophysiological features shared with TBI, potentially priming the brain for worse outcomes [39]. Furthermore, individuals with PTSD may exhibit maladaptive behavioural responses to stress, triggering an overexaggerated emotional response to symptoms and a prolonged perceived severity of their injury [43].

Beyond structural damage, attentional bias to threat, a hallmark symptom of PTSD [44,45] may exacerbate PPCS or delay recovery by sustaining a heightened state of hyper- arousal [46]. Individuals who have experienced trauma can engage in hypervigilant threat monitoring, resulting in cognitive biases towards potential dangers [45,47], hypothesised to be mediated by increased amygdala activity [47], and increasingly recognized as a neurocognitive indicator of PTSD [48,49]. Research has shown that women IPV victim-survivors with PTSD exhibit dysregulated emotional responses to potentially threatening stimuli [50,51] and pain [52], and are biased towards IPV-related words [53]. Military veterans with PTSD have also been shown to catastrophize somatic and physical sensations [54] commonly endorsed by individuals with PPCS [9]. Taken together, victim-survivors with poor attentional control and heightened fear sensitivity due to PTSD may be hypervigilant towards brain injury symptoms [30] creating a self-reinforcing cycle whereby distress of unresolved PPCS further sustains elevated threat arousal [55]. Individuals may interpret normal post-concussion symptoms as dangerous, reinforcing avoidance and prolonging recovery.

Despite the high prevalence of PTSD and the possible increased risk of PPCS among victim-survivors, there remains limited standardised therapy addressing the chronic sequelae of IPV-BI. Here, we first identify intervention studies that specifically evaluate therapies for PTSD and PPCS in IPV victim-survivors. We then provide a narrative overview of therapeutic approaches used across intervention categories, including psychotherapy, mindfulness-based practices, exercise, and pharmacotherapy. As women are disproportionately affected by IPV, particular attention is given to interventions with demonstrated utility in populations of women. Given the limited IPV-specific literature, we also draw narratively on evidence from mixed-sex studies and from comparable populations, such as military and sporting groups where PTSD and brain injury are also prevalent, to contextualize and inform treatment approaches.

## 2. Methods

This review had two aims: to identify key therapeutic studies addressing PPCS and PTSD in IPV survivors and to provide a narrative overview of potential therapeutic categories, including psychotherapy, mindfulness and meditation, exercise, and pharmacotherapy. A narrative review format was selected, given the breadth and heterogeneity of the topic, and the limited IPV-specific evidence base.

We conducted a comprehensive literature search using PubMed and Google Scholar applying the terms ‘intimate partner violence’, ‘IPV’, ‘persistent post-concussion symptoms’, “post-concussion syndrome’, ‘PPCS’, ‘mild traumatic brain injury’, ‘mTBI’, ‘pharmacotherapy’, ‘exercise’, ‘psychotherapy’, ‘depression’, and ‘post-traumatic stress disorder’. Articles were eligible if they were full-text, peer-reviewed journal articles published in English. Inclusion parameters required that studies be conducted in women with a history of IPV and report primary or secondary treatment outcomes related to PPCS or PTSD. No date restrictions were applied. Nineteen key studies were identified and summarized in Table 1.

Four therapeutic categories were identified as the organisational framework for the narrative discussion: psychotherapy, mindfulness and meditation, exercise, and pharmacotherapy. These categories were selected based on their relevance to PTSD and PPCS management and the availability of literature in IPV and related populations. The 19 identified IPV-specific studies are integrated within the relevant sections of the narrative discussion. Where no IPV-specific evidence was identified for a given intervention category, relevant studies from closely related populations, including military veterans, athletes, and general TBI samples, were narratively reviewed to inform potential therapeutic implications for IPV-PPCS and PTSD.

## 3. Results and Discussion

Overall, 19 studies that addressed PTSD in women IPV survivors as either primary or secondary outcomes were identified. Table 1 overviews the intervention, sample population, study objectives, and primary and secondary outcomes of all studies. Notably, the identified studies predominantly utilized psychotherapeutic or meditation/mindfulness-based interventions. No interventions for IPV-PPCS were identified. A total of 14 out of 19 identified studies utilised forms of psychotherapy, either applying techniques previously used in PTSD or trauma-focused psychotherapy [56,57,58,59,60,61,62,63,64,65,66,67,68,69] such as cognitive behavioural therapy (CBT), cognitive-narrative therapy and cognitive processing therapy (CPT). Four studies combined psychotherapeutic approaches with meditation and/or mindfulness [70,71,72,73]. A single study assessed exercise intervention (i.e., virtual dance and movement workshops) [74]. No pharmacotherapeutic interventions targeted to PTSD or PPCS in IPV survivors were identified. Notably, the identified literature was methodologically diverse, comprising studies that vary considerably in sample size, outcome measures, follow-up periods, and intervention duration. Many studies were designed primarily to assess feasibility and acceptability, where treatment efficacy was a secondary outcome [57,70,72,73]. Due to this variability, cross-study comparisons should be interpreted with caution, and formal conclusions about comparative efficacy cannot be drawn from the existing evidence base.

With these limitations in mind, the following discussion provides a narrative overview of the existing and potential therapies across intervention categories (i.e., psychotherapy, mindfulness-based practices, exercise, and pharmacotherapy). Where relevant, 19 studies on IPV-PTSD have been integrated. Due to the lack of research specific to IPV, narrative insight will be drawn from therapeutic studies targeting co-morbid conditions in IPV (i.e., depression) and studies that assess PTSD or PPCS in closely related populations (e.g., military, sports-related concussions). At least a proportion of the study population of these supplemental studies included women. Importantly, given that no interventional studies specific to IPV-PPCS were identified, this discussion should be interpreted as translational and offer insight for hypothesis generation of future research.

### 3.1. Psychotherapy

Psychotherapies, conducted by a psychologist, psychiatrist, or counsellor, are common approaches to address PTSD, depression, and anxiety [75,76]. We identified 14 studies with demonstrated efficacy in addressing PTSD among IPV survivors, but none addressing PPCS in IPV survivors using a range of therapeutic approaches. While other psychotherapeutic approaches may be relevant, a comprehensive theoretical overview is beyond the scope of this review. For a detailed discussion, see Hameed et al., 2020 [77].

Cognitive behavioural therapy (CBT) is currently considered the “gold standard” psychotherapeutic approach [78], and has demonstrated efficacy for IPV survivors living with PTSD [56]. For example, survivors who participated in an 8-week CBT program supplemented by either exposure techniques or communication skills training experienced a decrease in PTSD, depression, and anxiety severity but saw no significant changes in self-esteem [56]. Modifications of CBT, such as cognitive-narrative therapy, Helping to Overcome PTSD through Empowerment (HOPE), and cognitive processing therapy (CPT), have also been developed for IPV survivors. Cognitive-narrative therapy involves modifying the narrative surrounding one’s traumatic experiences using individualised themes and objectives and was trialled in IPV survivors residing in protective housing with high satisfaction but low efficacy to reduce in PTSD, depression, and borderline personality disorder symptoms [57]. Similarly, HOPE was also designed to target PTSD in IPV survivors residing in shelters [58,59,60,61] and integrates Herman’s multistage model of recovery [79] and CBT and emphasises safety, empowerment, symptom management, and relationship improvement [59]. Preliminary randomized controlled trials found that HOPE reduced depression symptoms and revictimization rates and increased community service with effects persisting in 94.6% of participants at 6-month follow-up [59]. However, no improvement in PTSD severity was observed when compared to standard shelter services [59,61]. A subsequent trial with post-shelter HOPE sessions demonstrated significant PTSD reduction and higher employment rates at follow-up, suggesting that IPV survivors require continued support beyond shelter discharge.

CPT, originally developed for survivors of childhood and adult sexual violence, has been extended for IPV-related PTSD is a recommended treatment by the American Psychological Association and the Veterans Health Administration and Department of Defence [76]. Among IPV survivors of physical assault, CPT reduced revictimization likelihood at 6-month follow-up, even among women who remained in abusive relationships [63]. Among female rape survivors, CPT remediated PTSD, depression, and guilt compared to waitlisted women [62], with gains potentially sustained for 5–10 years [80]; however, the outcomes were comparable to prolonged exposure treatment. It was determined that while both the cognitive component and written component of CPT improved PTSD and depression, though the cognitive component yielded slightly faster remediation [64]. While expressive writing has shown utility for cognitive reconstruction in IPV survivors [65], this population may benefit from more structured, guided interventions. Notably, CPT can be delivered by phone, reducing barriers for rural and remote populations [81] and requires nearly half the work from participants compared to exposure therapy, suggesting that it may be a more efficient option [62]. While HOPE, CPT, and exposure therapy have each demonstrated reductions in PTSD symptomology across varying study contexts, the complex and heterogeneous nature of IPV survivors’ experiences may require a more trauma-focused approach [77,82,83]. Cognitive trauma therapy for battered women (CTT-BW) is a CBT adaptation targeting the multimodal nature of IPV trauma, focusing on assertiveness, self-advocacy, and revictimization prevention [66,82]. Pilot studies reported PTSD remittance in 94% of participants and 60–72% reductions in depression, guilt, and shame [66], with a subsequent trial reporting similar benefits in 87% of participants at 6-month follow-up [67]. CTT-BW has demonstrated neuronal reorganisation in the circuitry responsible for responses to trauma-related stimuli and thus may improve emotional dysregulation and hyperreactivity [68]. Importantly, CTT-BW can be delivered without extensive mental health training [69], improving accessibility for women without financial means or who prefer community-based care. Taken together, psychotherapy shows considerable promise for IPV-related PTSD; however, the heterogeneity of the existing evidence in terms of setting, sample, and design (highlighted in depth Table 1) warrants caution. Large-scale validation studies or meta-analyses are needed prior to adoption in clinical practice.

Psychotherapy has also been applied to manage PPCS symptoms in adolescents with PPCS due to sports-related concussions. Although this remains unstudied in IPV survivors, insights from non-IPV mTBI populations may inform adaptations of these approaches. Firstly, the perceptions of diagnosis and prognosis in the early stages of mTBI patient recovery can affect symptom onset and severity, regardless of injury severity, with poor perceptions of diagnosis potentially contributing to the development of PPCS [84]. Psychoeducation addressing these perceptions, including symptom information booklets, coping strategies, and follow-up assessments, significantly reduce PPCS risk [85,86], whereas the effectiveness of CBT to manage PPCS risk has shown mixed results [87]. Whether psychotherapies such as CBT may improve PPCS within IPV survivors is, however, not known.

Overall, psychotherapy was found to be the most highly researched therapeutic area among IPV survivors and shows promise; however, it is not always accessible [77,88]. The average number of sessions needed for recovery is 9.5, but the range can be from 2 to 50, depending on symptom severity [77]. Furthermore, long wait times and financial burdens present considerable barriers, particularly for marginalised and low-income populations who experience disproportionately higher rates of IPV [89]. Traditional psychotherapies may not be culturally appropriate for all individuals, and cultural differences should be acknowledged when considering appropriate interventions. Finally, trauma-focused psychotherapy may carry the risk of traumatization and contribute to high dropout rates; prioritizing trauma-informed care is essential. Combining psychotherapy with exercise or pharmacotherapy may offer additional benefits [90].

### 3.2. Mindfulness and Meditation

Mindfulness and meditation-based interventions are becoming increasingly popular as therapeutic avenues for trauma and PTSD and is therefore of interest in survivors of IPV. While these styles of approaches have shown success in mediating physical and mental health problems such as PTSD, depression, and anxiety disorders unrelated to IPV [73], efficacy in IPV survivors remains understudied. Here, we identified one study that designed a psychomotor therapy called Feel–Own–Move (FOM) which utilises mindfulness and meditation principles to help women who have experienced IPV residing in shelters become “in tune” with their body through deliberate movements and bodily awareness, a sense which, when dampened, is hypothesized to be a factor implicated in PTSD, depression, and anxiety [73,91]. The core principles of FOM are built upon bodily awareness and self-regulation through movement, drawing, and verbal and non-verbal communication [73]. FOM was found acceptable, yielding 71% retention and high satisfaction. Preliminary efficacy results found significantly decreased levels of bodily dissociation post-treatment, and a large effect size on environmental quality of life [73]. However, there were no significant differences between pre- and post-treatment PTSD, depression and anxiety symptoms [73]. While larger-scale trials are needed to confirm the therapeutic utility, alternative mindfulness interventions such as mindfulness-based stress reduction (MBSR), while not specific to PTSD, have demonstrated beneficial therapeutic outcomes. MBSR delivered in group settings has been qualitatively reported to reduce stress and increase empowerment, self-compassion, and interpersonal skills in women with IPV histories. The group setting reportedly reduced fear of judgment and promoted self-compassion and emotional regulation, however quantitative confirmation of treatment efficacy for PTSD is needed [92,93]. Interpersonal Therapy and Forgiveness Therapy integrate modern psychotherapy and mindfulness techniques to strengthen interpersonal skills and social support and have reduced PTSD symptoms in pregnant IPV survivors and in women living in shelters [70,71,72]. MBSR and Interpersonal Therapy are also highly accessible and acceptable to underrepresented populations with demonstrated utility in minority and low-income groups. Overall, mindfulness and meditation-based interventions are useful, particularly when clinical care, such as psychotherapy, cannot be accessed consistently (i.e., cost or inaccessible to rural minorities). However, many of the existing studies are preliminary in nature, with feasibility and acceptability as primary outcomes; further large-scale efficacy studies are needed. Further, many psychotherapeutic approaches including CBT already incorporate mindfulness elements and while they have been addressed separately in this review may be most effective when used in combination particularly when facilitated by a clinician to ensure personalized, trauma-informed care. With respect to PPCS, while no IPV-specific literature exists, evidence from a meta-analysis of clinical, athletic, and veteran populations suggests mindfulness and MBSR may improve mTBI-related fatigue, depression, and quality of life [94]. However, IPV-specific research is needed to confirm the applicability of these findings.

### 3.3. Exercise

Exercise is well known to improve symptoms of PTSD and depression in populations with high rates of gendered violence [95,96,97] and to mitigate PPCS [98,99], making it a promising therapeutic avenue for IPV survivors [98,99,100,101,102]. Despite this, the literature specific to this population remains limited; we only identified a single feasibility study using exercise to mitigate PTSD symptoms in IPV survivors. Move to Move Beyond, a 12-session virtual dance and creative movement workshop delivered during COVID-19, yielded lower PTSD symptoms and psychological stress compared to controls, and participants reported feeling greater connection with their body and sense of community [74]. While not specific to PTSD, the Through Every Mile (STEM) 10-week community-based running program improved depression, anxiety, and overall quality of life in IPV survivors and may translate to PTSD, given considerable symptom overlap [103]. Similarly, yoga, and specifically trauma-informed yoga, combining low-intensity physical movements and meditative techniques, has significantly reduced stress and improved self-efficacy among IPV survivors [104,105] with transferrable breathing and relaxation techniques that women can use outside of guided sessions [106]. Outside of IPV populations, trauma-informed yoga has also led to higher remission rates for PTSD, depression, and anxiety compared to health education alone in women [107,108] and has higher completion rates than CPT [109]. While trauma-informed yoga may offer an avenue to address PTSD in IPV survivors, clinically meaningful evidence to reduce PTSD symptoms remains mixed [110,111] and has not been compared to traditional yoga in this population, which may offer similar benefits.

While no IPV-specific PPCS exercise studies exist, findings from sports-related concussion samples offer a preliminary theoretical basis for exercise benefits in this population, informing future hypotheses for clinical investigation. In this population, sub-symptom aerobic exercise, which involves exercising below the heart rate threshold that exacerbates concussion symptoms, is accepted as an effective intervention for promoting concussion recovery and preventing PPCS. Daily sub-symptom exercise has been shown to significantly improve symptom resolution and return to play compared to those assigned to passive stretching or standard care [112,113,114]. Within adults with PPCS, 74% whom were female, also significantly improved symptom burden, quality of life, and depressive symptoms [115]. While the results are promising, sub-symptom aerobic exercise treatment has been largely assessed only during acute and sub-acute injury recovery in otherwise healthy adolescents or young adults [116,117], with limited consideration given to pre- or post-injury mental health and no existing protocols are specific to IPV. Furthermore, practical barriers that may limit translatability within IPV survivors include extracranial injuries that affect the ability to exercise, or issues of housing or childcare that take precedence over a daily exercise routine. Therefore, aerobic exercise may be better suited for survivors who are no longer in a crisis setting (i.e., chronic time frame after injury); however, there is currently no consensus on a standardized treatment protocol. While a couple of published protocols exist [118,119], further evidence is needed to determine the optimal exercise intensity, type, frequency, and duration, for managing chronic PPCS [120]. Beyond efficacy, ensuring a trauma-informed approach is essential for translation into IPV populations [102,121]. For example, feeling safe during exercise, preferring women-led, trauma-informed programs that minimize focus on appearance, avoid non-consensual touch, and offer flexible, at-home formats such as yoga, walking, or app-based coaching are preferred [102,121,122]. Furthermore, individualised approaches were found to support adherence and should be considered when translating approaches to survivors of IPV.

### 3.4. Pharmacotherapy

Pharmacotherapies are commonly used to treat mental health conditions; however, despite evidence that women with a history of IPV are more likely to use psychotropic medication than women without IPV exposure [123,124,125,126], no studies, to our knowledge, have investigated pharmacotherapies specifically for IPV-related PTSD or PPCS. As such, the following discussion draws on evidence from closely related populations to highlight potential pharmacotherapeutic avenues for future investigation in IPV survivors. Selective serotonin reuptake inhibitors (SSRIs) are first-line treatment options for depression regardless of illness origin, and their efficacy has been explored in PTSD [127] and mTBI-related depression [128] with moderate success. While not specific to IPV, the closest available evidence comes from a pilot trial of risperidone monotherapy [129] among women who had experienced sexual assault or domestic violence and two case studies in family violence of adjunctive brexpiprazole [130], both showing reductions in PTSD symptoms, though small sample sizes limit generalisability. Sertraline and Paroxetine are approved by the FDA for the treatment of PTSD [127,128], and while meta-analyses have demonstrated a significant overall reduction in PTSD symptoms due to SSRIs, they were not as effective as trauma-focused therapy [131]. SSRIs alone however, may not be the most appropriate first-line treatment for PTSD compared to combination therapy of psychotherapy and pharmacotherapy [131]. Notably, nearly a third of PTSD patients are suggested to be treatment-resistant [132], and additional pharmacotherapies (e.g., tricyclic antidepressants, antipsychotics, and benzodiazepines) have had limited success [133]. Non-adherence to medication regimens in IPV survivors has been documented with financial barriers, reluctance or forgetting to take medication as common reasons [134,135,136]. In many instances, pharmacological interventions for PTSD and PPCS are limited to symptom management, rather than resolving the underlying issue. For example, sleep disturbances, common to both mTBI and PTSD [137,138], may benefit from low doses of melatonin, which may offer neuroprotection against oxidative stress [139,140], or insomnia medications for temporary relief [141]. Similarly, post-traumatic headaches, which is consistently the most common persisting symptom across TBI severities [142], can be addressed with over-the-counter analgesics (e.g., acetaminophen, nonsteroidal anti-inflammatory drugs). Novel therapeutics that move beyond symptom management and target the neurobiological mechanisms underlying both PTSD and PPCS are needed.

One emerging avenue is the renewed interest in classical psychedelics; however, it should be noted that evidence in this area is preliminary, largely derived from non-IPV populations, and its relevance to IPV survivors remains uncertain. Classical psychedelics include psilocybin and lysergic acid diethylamide (LSD), while non-classical psychedelics include ketamine and 3,4-methylenedioxymethamphetamine (MDMA). Both have demonstrated utility as therapeutic interventions when used in combination with psychotherapy. Phase III clinical trials of MDMA-assisted psychotherapy in patients with combat and multiple traumas showed that 67–71% of participants no longer met the diagnostic criteria for PTSD, with no drug-seeking behaviours reported [143,144]. While not specific to IPV, a small cohort study with low-dose MDMA reduced chronic treatment-resistant PTSD in women who had survived sexual assault [145]. Similarly, ketamine reduced PTSD and depression symptoms within 24 h of dosing in a sample (59% women) with PTSD due to sexual or physical assault [146]. Notably, psychedelic-assisted therapy has demonstrated greater efficacy than both non-drug therapy [143,147] and conventional pharmacological interventions [148,149] for PTSD, depression, and substance use disorders in the general population, though its relevance to IPV survivors remains to be established. A theoretical basis for psychedelic utility in TBI and PPCS has been proposed [129]; however, empirical evidence is similarly sparse. One existing study found that ibogaine significantly decreased PTSD, depression, and anxiety symptoms immediately after treatment and at 1-month follow-up in male veterans with chronic mTBI [150]. The potential role of classical and non-classical psychedelics as pharmacotherapies for PTSD and PPCS is still in its infancy, and whether preliminary evidence translates into populations of IPV survivors remains to be determined. Optimal dosages, the most effective route of administration, and the safety of long-term use are still unknown [148]. Furthermore, marginalised and low-income groups, common among IPV survivors, are underrepresented in psychedelic research [151,152]. Taken together, while pharmacotherapy remains commonly used for PTSD, it is ineffective for many. Psychedelics, particularly in combination with psychotherapy, are a promising alternative, though research remains preliminary, and the safety, feasibility, and efficacy of psychedelic-assisted therapy among IPV survivors are not known.

## 4. Limitations and Future Directions

This review has a number of limitations. Firstly, there were only 19 studies to draw any conclusions on the effectiveness of four different treatment modalities, specifically in IPV victim-survivors, necessitating the addition of narrative insights from military veterans and sports-related concussion literature to inform the discussion. Furthermore, the identified studies were methodologically heterogeneous, with many designed primarily to assess feasibility and acceptability rather than treatment efficacy. While many of the reviewed interventions show promise, future large-scale clinical trials are needed to confirm their effectiveness and draw definitive conclusions about efficacy. Finally, as the included studies focused predominantly on women IPV survivors, future research should consider the therapeutic needs of non-women populations.

The mental health sequelae that often follow IPV are vast; therefore, the decision to focus solely on treatments for PTSD and PPCS disregards therapeutic options targeting other mental health conditions (i.e., depression or anxiety) common in this population. However, there are a number of reviews which go into depth in this area. Lastly, there are no treatment avenues that aim to target both PTSD and PPCS in IPV survivors, war veterans, or individuals who have sustained a sports-related concussion. While it is important to understand how to manage each of these conditions individually, realistically, they both often occur in tandem and have considerable symptom overlap. The precise nature of the PPCS-PTSD overlap in IPV survivors remains poorly understood, and given the high rates of comorbidity in this population, understanding this intersection is an important priority for future research. Further work is needed to better understand the potential synergistic effects of these conditions and develop integrated treatment approaches.

A common theme for all interventions is the need to conduct larger-scale and appropriately designed and controlled clinical trials for the unique setting of IPV. Trauma-informed care and themes of inclusivity, safety, and empowerment is widely emphasized by research experts and lived experience advocates [153,154,155,156]. Trauma-informed care has shown modest but statistically significant symptom improvement compared with treatment as usual [157] and has been associated with greater empowerment, reduced social withdrawal, and improved emotional regulation [158]. While trauma-informed strategies are primarily discussed in the context of clinical care and practitioner education, these principals are essential to guide the design and conduct of clinical trials [159]. Participant autonomy within research participation has been particularly emphasized to improve adherence and prevent retraumatization. For example, researchers may implement a preference for physical activity in exercise trials [122] or increase flexibility for participation to accomodate transient housing or childcare commitments that hinder consistent availability [59,60].

Finally, there is a large gap in practice and literature surrounding women who remain in their abusive relationships. Treatments for survivors removed from the IPV setting may not be generalizable to those who remain in the crisis setting; alternative interventions promoting personal safety, legal options, reassurance, and addressing harmful coping mechanisms, however, are the priority [83,160].

## 5. Conclusions

IPV may put women victim-survivors at a high risk for PPCS and PTSD [4,161]. Current evidence on therapeutic options addressing these outcomes in IPV survivors remains limited, highlighting the need for further research into prevention, intervention, and care specific to this population. We identified 19 studies assessing therapeutic interventions for PTSD in IPV survivors, predominantly psychotherapies. In contrast, no studies were identified that addressed PPCS in this population. As such, potential therapeutic interventions for IPV-related PTSD and PPCS across psychotherapy, mindfulness-based practices, exercise, and pharmacotherapy were discussed narratively. However, given that these discussions were drawn from military or sporting contexts, suggestions remain largely inferential, highlighting the need for empirical validation. There is also a lack of research addressing the complex intersection of PTSD and PPCS. Future interventions should integrate trauma-informed and accessible approaches that consider combination therapies to address the complexities, such as the potential synergistic effects of PPCS and PTSD, within this population.

## Figures and Tables

**Table 1:** Summary of studies specifically investigating therapeutics for PTSD in women IPV survivors.

	Intervention	Sample Population	Objectives	Primary Outcomes	Secondary Outcomes	Reference
Psychotherapy	Cognitive behavioural program with exposure techniques or communication skills training (1-, 3-, 6- and 12-month follow-up post-shelter)	n = 53 (exposure techniques = 28; communication skills training = 25); Age = 41 +/− 9.26	Efficacy study: evaluated the efficacy of cognitive behavioural therapy with exposure therapy or communication skills training in reducing mental health symptoms	Decrease in PTSD (Severity of PTSD symptoms scale), depression (BDI), and anxiety (BAI) symptoms in both treatment groups	Decrease in anger (STAXI-2) over time in exposure group but not statistically different within subjects; no significant increases in self-esteem (RSES) or decreases in anger levels (STAXI-2)	Crespo & Arinero, 2010, [56]
Psychotherapy	Cognitive-narrative therapy (four 60-min sessions per week with a different focus each session)	N = 23 women recruited from a protection shelter house (Treatment group = 12; Control group = 11); TG age = 37 +/− 13.57, CG age = 41.82 +/− 10.89	Efficacy study: evaluated the ability of cognitive-narrative therapy to reduce mental health symptoms	Positive but insignificant differences in depression between groups (PHQ-9)	Positive but insignificant PTSD effect between treatment and SSS; C-PTSD (ITQ); high levels of program satisfaction (IASPI)	Moreira et al., 2022 [57]
Psychotherapy	HOPE (9–12 sessions, 2 sessions per week; 1-week, 3- and 6-months follow-up)	n = 18; Age = 32 +/− 7; White = 9, African-American = 7, Hispanic = 2, Other = 2	Pilot study: evaluated the initial feasibility and efficacy of HOPE	High treatment satisfaction with minimum dose (CSQ)	Decreased PTSD (CAPS) and depression (BDI) symptoms, social impairment, and resource loss (COR-E); increase in effective use of resources (EOR); gains were maintained up to 6 months post-shelter	Johnson & Zlotnick, 2006 [58]
Psychotherapy	HOPE or standard shelter services (12 sessions over 8 weeks; 1-week, 3- and 6-months follow-up)	n = 70 (HOPE n = 35; Standard Shelter Services (SSS) n = 35); Age = 32.55 +/− 8.00; African American n = 35, Caucasian n = 30, Other n = 5, Hispanic n = 3	Randomized clinical trial: compared the acceptability, feasibility, and initial efficacy of HOPE to SSS	Decreased emotional numbing, effortful avoidance, and arousal PTSD symptoms (CAPS) over time but no significant differences in overall PTSD severity; less likely to report re-abuse (CTS2) at 6-month follow-up	Large effect size in reduction of depression symptoms (BDI); empowerment (PPS-R) and social support; no differences in resource loss (COR-E); high satisfaction rates (CSQ) and low (6.67%) dropout rate	Johnson et al., 2011 [59]
Psychotherapy	HOPE or standard shelter services (16 sessions over 8 weeks, 10 sessions in shelter, 6 sessions post-shelter; 1-week, 3- and 6-month follow-up)	n = 60 (HOPE n = 30; Standard Shelter Services n = 30); Age HOPE = 33.30 +/− 10.48, Shelter age = 33.20 +/− 10.39; African American n = 34, Caucasian n = 26, Hispanic n = 4	Randomised clinical trial: Compared the acceptability, feasibility, and initial efficacy of an expanded version of HOPE to SSS	Less severe PTSD (CAPS) symptoms at 6-month follow-up; no difference in revictimization (CTS2); only 2 women dropped out	Less severe depression (BID) symptoms at 6-month follow-up; increased feelings of empowerment (PPS-R), gain of personal and social resources (COR-E), and more likely to be employed at 3- and 6-month follow-up; no changes in satisfaction of social support (SSQSF)	Johnson et al., 2016 [60]
Psychotherapy	HOPE or PCT+ (16 sessions over 8 weeks, 10 sessions in shelter, 6 sessions post-shelter; 6- and 12-month follow-up)	n = 172 (HOPE n = 83; PCT+ = 89); HOPE age = 34.34 +/− 9.46, PCT+ age = 35.87 +/− 8.78; Black/African American n = 76, White n = 80, Multiracial n = 15)	Randomised clinical trial: Compared efficacy of mental health symptoms reduction of HOPE to an adapted version of PCT+	Significant reductions in PTSD (CAPS) and IPV (SVAW and PMWI-SF), with no differences between treatment groups, although many participants still met clinically significant symptoms; all differences were maintained at 12-month follow-up	Significant improvements in depression (CES-D), health-related quality of life (MCS), empowerment (PPS-R), and post-traumatic cognition (PTCI); high satisfaction for both treatments (CSQ-8)	Johnson et al., 2020 [61]
Psychotherapy	CPT, CPT-C or WA (6-week intervention, 12 h total of therapy; 6-month follow-up)	n = 150 (CPT n = 53; Cognitive only CPT n = 47, Written account only n = 50); Age = 35.4 +/− 12.4; Caucasian = 62%, African American = 34%, “Other” = 4%	Efficacy study: Evaluated the ability of CPT to reduce mental health symptoms and risk of IPV revictimization. Addtional analysis of [62]	Significantly decreased PTSD (PDS) & depression (BDI-II) symptoms	Revictimization: women who responded well to treatment reported less IPV at 6-month follow-up	Iverson et al., 2011 [63]
Psychotherapy	CPT, PE, or MA (two 60–90 min session per week, for a total of 13 h of treatment)	n = 121 (CPT = 41, PE = 40, MA = 40); Age = 32 +/− 9.9; White = 71%, African American = 25%, Other = 4%)	Efficacy study: Compared the ability of CPT to reduce mental health symptoms with PE and MA	Both CPT and PE decreased PTSD (CAPS) depression (BDI) symptoms, but CPT was more efficient; PTSD improvement in CPT group was maintained at 9-month follow-up	CPT remediated more guilt symptoms than PE (TRGI)	Resick et al., 2002 [62]
Psychotherapy	CPT, CPT-C, or WA (2 h of therapy per week for 6 weeks; 2-week and 6-month follow-ups)	n = 150 (CPT = 53, CPT-C = 47, WA = 50); Age = 35.4 +/− 12.4; White = 62%, African American = 34%, Hispanic = 3%, Other = 4%	Dismantling study of CPT: Compared treatment efficacy of a full CPT protocol to its cognitive (CPT-C) and written (WA) components	All treatments significantly decreased PTSD (PDS) and depression (BDI-II) symptoms; between groups, CPT-C performed better than WA; changes were maintained at 6-month follow-up	All treatments improved anxiety (STAI), anger (STAXI), shame (ESS), guilt (TRGI), dysfunctional cognitions	Resick et al., 2008 [64]
Psychotherapy	Expressive writing (20 min sessions, one session per week for 4 weeks; 4-month follow-up)	n = 59 (expressive writing = 25; neutral writing control = 22); Age = 36.5 +/− 8.9; White/European American = 68%, Latina/Hispanic = 13%, Middle Eastern = 6%, African American = 6%, Asian American = 2%, Other = 4%	Efficacy study: Compared the ability of expressive writing to reduce mental health symptoms with neutral writing	No significant reduction in depression symptoms (BDI) compared to control group	No change in PTSD symptoms (PCL-5) or pain (bodily pain scale of SF-36)	Koopman et al., 2005 [65]
Psychotherapy	CTT-BW (7–10 sessions (avg = 8.5); 3-month follow-up)	n = 37 (Immediate n = 19, Delayed n = 18, Dropout n = 5); Age = 36.4 +/− 9.1; White n = 18, Asian n = 10, Pacific Islander n = 6, Other n = 3	Pilot study: Evaluated the treatment effects of CTT-BW on mental health symptoms	PTSD (CAPS): Remitted PTSD Criterion C symptoms in 94% women and maintained at 3-month follow-up	Depression (BDI) scores returned to normal and maintained at 3-month follow-up; reduced feelings of guilt (TRGI), shame, and negative self-esteem (RSES); high treatment compliance and participant satisfaction (CSQ-8)	Kubany et al., 2003 [66]
Psychotherapy	CTT-BW (number of sessions = 9.5 +/− 1.6; 3- and 6-month follow-up)	n = 125 (Completers n = 86, non-completers n = 21, Dropout n = 5); Age = 42.2 +/− 10.1; White = 66, Native Hawaiian n = 11, Filipino n = 9, Japanese n = 8, Black = 6, Samoan n = 6, American Indian n = 2, Other/Mixed race n = 17	Efficacy study: Evaluated the ability of CTT-BW to reduce mental health symptoms	Remitted PTSD (CAPS, DEQ) in 87% of participants and largely reduced depression symptoms (BDI) at both follow-ups; TRGI	Slight increases in self-esteem (RSES), maintained at both follow-ups; large reductions in guilt (TRGI) that were maintained at both follow-ups	Kubany et al., 2004 [67]
Psychotherapy	CTT-BW (number of sessions = 11.57 +/− 1.60; 3-month follow-up)	n = 14 (dropout n = 6); Age = 40.07 +/− 7.44	Efficacy study using fMRI: Examined effects of CTT-BW on amygdala, insula, and cingulate responses during cognitive tasks as well as mental health symptoms	MRI scan: Decreased cortical response during anticipation and presentation of emotionally triggering images	Decreased PTSD (CAPS & PCL) and depression (BDI-II) scores post-treatment and maintained at follow-up	Aupperle et al., 2013 [68]
Psychotherapy	CTT-BW (11–12 weeks, 1 session/week; 1-month follow-up)	n = 8; Age = 28–56; Caucasian n = 5, African American n = 2, Mixed-race n = 1	Replication study: Replication and extension of [67]	Large effect sizes between pre- and post-treatment PTSD (CAPS) and depression (BDI-II) scores, which were maintained at follow-up	Decreased anxiety (BAI) scores post-treatment and maintained at follow-up; self-esteem (RSES) and quality of life (QOLI) significantly improved post-treatment and at follow-up	Beck et al., 2016 [69]
Mindfulness and Meditation	FT or AT (1 session/week for 5–12 months)	n = 20 (FT = 10; AT = 10); Age = 44.95 +/− 7.01; European Americans n = 18, Hispanic American n = 1, Native American n = 1	Efficacy study: Compared the ability of FT to reduce mental health symptoms with AT	Reduction in depression (BDI-II), trait anxiety (STAI), and PTSD symptoms (PTSS checklist) in FT group; increase in self-esteem in FT group (CSEI)	Improvements in forgiveness (EFI) and finding meaning in suffering in FT group	Reed & Enright, 2006 [70]
Mindfulness and Meditation	Community-based IPT (1 session/week for 8 weeks; 1-week and 3-month follow-up)	n = 32; Age = 39.8 +/− 9.7; African American n = 16, White n = 13, American Indian n = 1, Multiracial n = 1, Hispanic n = 1	Pilot study: Examined the feasibility, acceptability, and preliminary benefits of IPT	Feasibility: 65.6% of women attended at least one session and average attendance was 5.9 sessions; acceptability: subjective treatment satisfaction was high in most participants	Decreased depression (HRSD) and PTSD (MPSS—frequency and severity) symptom severity and improved interpersonal functioning (IIP-32)	Cort et al., 2014 [71]
Mindfulness and Meditation	IPT for pregnant victim-survivors (1 session/week for 4 weeks + 1 session within 2 weeks of delivery; follow-ups 5–6 weeks after intake, 2 weeks post-delivery, 3-months postpartum)	n = 54 (IPT n = 28; Control n = 26); Age = 23.8 +/− 4.6; Hispanic = 42.6%; White = 38.9%; Black = 11.1%, Other/Multiracial = 7.4%	Pilot study: Examined feasibility, acceptability, and efficacy of IPT specifically in pregnant victim-survivors	No decrease in major depressive disorder episodes and PTSD (LIFE; moderate decrease in depression (EPDS) and PTSD (DTS) symptoms during pregnancy and 3 months postpartum	Feasibility and acceptability: average attendance rate of 3/5 appointments suggesting intervention was acceptable to most participants; no change in IPV revictimization (CTS2)	Zlotnick et al., 2011 [72]
Mindfulness and Meditation	Feel–Own–Move (8-week intervention; 3 sessions/week; 24 session total)	n = 17 (Age = 42.8 +/− 11.1)	Feasibility study: Evaluated feasibility, efficacy, and preliminary intervention effects of FOM	Feasibility: 94% of women invited to participated accepted with a 71% retention rate; participants attended 86% of individual and 75% of group therapy sessions; acceptability: high program acceptability and satisfaction using a survey designed by the authors	Decreased levels of bodily dissociation (SBC) and increased willingness to engage in activities; no significant changes in PTSD (PCL), anxiety and depression (HADS), or quality of life (WHOQoL)	Machorrinho et al., 2023 [73]
Exercise	Move to Move Beyond (2 virtual sessions/week; 12 sessions in total over 6 weeks)	n = 45 (Intervention = 25; Control = 20); age = 23–48; over three quarters of participants identified as BIPOC	Feasibility study: Evaluated feasibility and initial mental and general health treatment effects of Move to Move Beyond	Program was enjoyable and had benefits in connection to the self and body, expression through movement, community building, relaxation, positive emotions, and applying self-care habits to daily life	Decrease in PTSD (PCL-5) symptoms overall, no difference between treatment and control; no changes in psychological distress (K6+); no change in heart rate variability	Özümerzifon et al., 2022 [74]

AT = alternative treatment; BAI = Beck Anxiety Inventory; BDI = Beck Depression Inventory; BIPOC: Black, Indigenous, and people of colour; CAPS = Clinically-Administered PTSD Scale; COR-E = The Conservation of Resources Evaluation; CPT = Cognitive Processing Therapy; CSEI = Coopersmith Self-Esteem Inventory; CSQ-8 = Client Satisfaction Questionnaire; CTS2 = The Revised Conflict Tactics Scale; CTT-BTW = Cognitive Trauma Therapy for Battered Women; DEQ = Distressing Event Questionnaire; DTS = Davidson Trauma Scale; EFI = Enright Forgiveness Inventory; EPDS = Edinburgh Postnatal Depression Scale; FT = Forgiveness Therapy; HOPE = Helping to Overcome PTSD through Empowerment; HRSD = Hamilton Rating scale for Depression; IIP = Inventory for Interpersonal Problems; IPT = Interpersonal Therapy; ISSB = The Inventory of Socially Supported Behaviours; K6+ = Kessler Psychological Distress Scale LIFE = Longitudinal Interval Follow-up Examination; MBSR = Mindfulness-Based Stress Reduction; MPSS = Modified PTSD Symptom Scale; PCL = PTSD checklist; PDS = Posttraumatic Diagnostic Scale; PPS-R = The Personal Progress Scale–Revised; PTSS checklist = Post-traumatic stress symptom checklist; QOLI = Quality of Life Inventory; RSES = Rosenberg Self-Esteem Scale; STAI = State-Trait Anxiety Inventory; STAXI-2 = Anger Expression Subscale from the State-Anger Expression; TRGI = Trauma-Related Guilt Inventory.

## Data Availability

No new data were created or analyzed in this study.
